# An Analysis of Endocannabinoid Concentrations and Mood Following Singing and Exercise in Healthy Volunteers

**DOI:** 10.3389/fnbeh.2018.00269

**Published:** 2018-11-26

**Authors:** Nicole L. Stone, Sophie A. Millar, Philip J. J. Herrod, David A. Barrett, Catharine A. Ortori, Valerie A. Mellon, Saoirse E. O’Sullivan

**Affiliations:** ^1^Division of Medical Sciences and Graduate Entry Medicine, School of Medicine, University of Nottingham, Nottingham, United Kingdom; ^2^Centre for Analytical Bioscience, Advanced Materials and Healthcare Division, School of Pharmacy, University of Nottingham, Nottingham, United Kingdom; ^3^BBC Studios “Trust Me I’m a Doctor”, BBC Scotland, Glasgow, United Kingdom

**Keywords:** endocannabinoids, anandamide, human, clinical, high, mood, singing and dancing

## Abstract

The euphoric feeling described after running is, at least in part, due to increased circulating endocannabinoids (eCBs). eCBs are lipid signaling molecules involved in reward, appetite, mood, memory and neuroprotection. The aim of this study was to investigate whether activities other than running can increase circulating eCBs. Nine healthy female volunteers (mean 61 years) were recruited from a local choir. Circulating eCBs, haemodynamics, mood and hunger ratings were measured before and immediately after 30 min of dance, reading, singing or cycling in a fasted state. Singing increased plasma levels of anandamide (AEA) by 42% (*P* < 0.05), palmitoylethanolamine (PEA) by 53% (*P* < 0.01) and oleoylethanolamine (OEA) by 34% (*P* < 0.05) and improved positive mood and emotions (*P* < 0.01), without affecting hunger scores. Dancing did not affect eCB levels or hunger ratings, but decreased negative mood and emotions (*P* < 0.01). Cycling increased OEA levels by 26% (*P* < 0.05) and tended to decrease how hungry volunteers felt, without affecting mood. Reading increased OEA levels by 28% (*P* < 0.01) and increased the desire to eat. Plasma AEA levels were positively correlated with how full participants felt (*P* < 0.05). Plasma OEA levels were positively correlated with positive mood and emotions (*P* < 0.01). All three ethanolamines were positively correlated with heart rate (HR; *P* < 0.0001). These data suggest that activities other than running can increase plasma eCBs associated with changes in mood or appetite. Increases in eCBs may underlie the rewarding and pleasurable effects of singing and exercise and ultimately some of the long-term beneficial effects on mental health, cognition and memory.

## Introduction

The classic “runners high” is described as the sense of well-being and mood elevation associated with moderate distance running. Other typical indicators include a decrease in anxious thinking (anxiolytic), positive emotions/mood (euphoria), reduced pain perception (analgesia) and a feeling of increased endurance (Sparling et al., 2003; Dietrich and McDaniel, 2004; Tsatsoulis and Fountoulakis, 2006; Raichlen et al., 2012). To explain these positive effects post-exercise, attention was directed to the endocannabinoid (eCB) system, and a number of groups have found significant correlations between physical activity, mood and elevated eCB levels. Interestingly, the majority of studies have only observed significant rises in the first identified eCB, anandamide (AEA; Sparling et al., 2003; Heyman et al., 2012; Raichlen et al., 2013), whilst the reports analyzing 2-arachidonylglycerol (2-AG) levels post-exercise have been less clear. Heyman et al. (2012) reported no change in circulating 2-AG levels after cycling. However, Brellenthin et al. (2017) showed that 2-AG and AEA were significantly increased in a study analyzing the effects of preferred (self-selected) and prescribed (70%–75% of max) exercise on eCB levels and mood.

The eCB system consists of the cannabinoid receptors 1 and 2 (CB_1_ and CB_2_), eCBs, and the enzymes that are responsible for their synthesis and breakdown (Devane et al., 1992; Mechoulam et al., 1995; De Petrocellis and Di Marzo, 2009). AEA and 2-AG are partial agonists of CB_1_ and CB_2_, whilst palmitoylethanolamine (PEA) and oleoylethanolamine (OEA) share similar synthesis and degradation mechanisms, without directly interacting with these receptors themselves (Hansen et al., 2000; Okamoto et al., 2004). Instead, these molecules interact with other receptors, primarily peroxisome proliferator-activated receptor alpha (PPAR-α) and transient receptor potential cation channel subfamily V member 1 (TRPV1; Ahern, 2003; Fu et al., 2003; Lo Verme et al., 2005a,b; Karwad et al., 2017). eCB signaling mediates a number of physiological and psychological processes including emotional responses, cognition, memory, motor behavior, feeding and energy consumption (Berger and Motl, 2000; Cota et al., 2003; Cota, 2007; Brellenthin et al., 2017). Studies have also established prominent roles of eCB signaling in the positive reinforcement in reward driven activities such as masturbation, arousal, binge-eating and social interactions in humans (Klein et al., 2012; Monteleone et al., 2015, 2017; Fuss et al., 2017).

Singing and dancing, especially as a group activity, are associated with positive mood in humans (Zajenkowski et al., 2015; Pearce et al., 2016; Tarr et al., 2016; Schladt et al., 2017). However, little has been studied to elucidate how these positive emotions are mediated. Recently, Hahn et al. (2017) studied the relationship between song practice and the eCB system in European starlings. They found a significant positive correlation between conditioned place preference (a measure of reward and song production), the number of songs a bird produced and the expression of CB_1_ in areas of the brain associated with reward, primarily the ventral tegmental area. Therefore suggesting a role for eCB signaling in singing and reward (Hahn et al., 2017; Riters et al., 2017). In humans, singing has been studied as a therapy for long-term disorders such as Alzheimer’s (to improve cognition, memory and long-term pain), chronic obstructive pulmonary disease, as well as to improve mood in conditions such as anxiety and depression (Reagon et al., 2016; Kang et al., 2017). Similarly, dancing has been explored as a potential therapy for cognitive and emotional dysfunction in conditions such as depression, dementia and Parkinson’s. In a systematic review of 11 studies, Kiepe et al. (2012) found that depression and psychological distress were reduced by dance therapy in patients suffering from Parkinson’s, diabetes, breast cancer or heart failure. Dance therapy in a group of 60 students also significantly reduced depression over a period of 12 weeks (Akandere and Demir, 2011). To date, no study has assessed singing or dancing and whether they modulate eCB levels in humans and whether that correlates to an improved mood. Given that mood is central in the measure of overall psychological well-being, low intensity activities that can positively modulate mood could be useful therapeutic tools in numerous conditions such as depression, anxiety and stress, especially if a patient cannot undertake moderate/higher intensity exercise.

The purpose of this study was to investigate whether activities other than running can give you a measurable “high” through changes in circulating eCBs levels. We examined activities that are associated with euphoria (singing and dancing) as well as an exercise regime other than running (cycling), with the hypothesis that these activities would increase plasma eCB levels. Quiet reading was used as a control condition. A secondary objective of this study was to establish whether there was a link between cycling, dancing, singing and reading with regards to mood and hunger ratings.

## Materials and Methods

### Participants

All procedures were approved by the University of Nottingham Faculty of Health Sciences ethics committee, and were carried out according to the declaration of Helsinki. Nine healthy post-menopausal female volunteers (age range 55–67, mean 61 years) were recruited from a local choir as people who enjoyed singing and exercise. The inclusion criteria were that volunteers be non-smokers, in good physical health, accustomed to singing in a group, and also enjoy exercise. Volunteers gave written informed consent prior to participation. The medications taken included antihypertensives (*n* = 2), antacids (*n* = 2), antidepressants/anti-anxiety medication (*n* = 2), HRT (*n* = 1), and an inhaler for asthma (*n* = 1).

Subjects arrived fasted (feeding affects plasma eCB levels; Monteleone et al., 2012) with no consumption of caffeine and this was verbally confirmed on arrival at the study facility. Participants were also asked to refrain from any exercise prior to attending the laboratory. Volunteers were unaware of the activity they were to perform on a given day until all baseline measurements were made to avoid any anticipatory effects.

### Study Days

Subjects came to the test site on four occasions between 8 am and 10 am in loose fitting sportswear. Each day, individuals were asked to complete two questionnaires before and after completing the activity. A visual analog scale (VAS) questionnaire was used to assess how hungry subjects were feeling on a scale of 1–10, using the questions “how hungry do you feel?”, “how full are you?”, “how much food could you eat?” and “how strong is your desire to eat?”. A positive and negative affect schedule (PANAS) questionnaire was used to assess subject’s mood before and after each activity using the following scoring system: 1 = “very slightly or not at all,” 2 = “a little,” 2 = “moderately,” 4 = “quite a bit” and 5 = “extremely;” Watson et al., 1988; Crawford and Henry, 2004). Positive affect score was calculated by adding the positive emotional responses and the negative affect score was calculated based on the addition of the negative affect scores.

Blood pressure was measured by oscillometry with the participant seated according to the British Hypertension Society guidelines, and heart rate (HR) was taken prior to commencing the activity and immediately after finishing the activity. Blood pressure and HR measurements were taken as the average over three (pre-activity) or 2 (post-activity) measurements. Blood draws (approximately 5 mL) were taken before commencing the activity and immediately after finishing the activity into pre-chilled K2-EDTA (Ethylenediaminetetraacetic acid) tubes and immediately placed on ice. After collection, blood was centrifuged at 2,000 *g* for 15 min at 4°C, plasma was removed and aliquoted, and immediately snap frozen in liquid nitrogen. Samples were stored at −80°C until subsequent analysis.

After the baseline measurements were made, volunteers were informed of the activity they were to perform. On day 1, volunteers did a supervised 30 min dance exercise class preceded by a 5 min warm up, to upbeat music. On day 2, volunteers did 30 min of supervised quiet reading (of boiler and dishwasher catalogs) to classical music. On day 3, volunteers for 30 min choir practice led by their choral director. On day 4, volunteers did a 30 min spin class (cycling) with a qualified instructor from the University of Nottingham Sports facility, with a 5 min warm up to upbeat music. All activities were performed as a group.

### eCB Quantification

eCB analysis was based on the method as described by Richardson et al. (2007). Samples were thawed and 100 μL of internal standard of 2-AG-d8 (10 μM) and 15 μL of AEA-d8 (28 μM) were added to a 0.4 mL aliquot of each plasma sample or blank sample (0.4 mL water) vortexed briefly. Ethyl acetate:hexane (9:1 v/v) was added to each sample and subjected for a slow vortex (10 min) and centrifuged for 13,000 rpm, 10 min, 4°C. The supernatants were transferred and the procedure was repeated. Supernatants were then pooled and evaporated using a centrifugal evaporator. Prior to analysis, each sample extract was reconstituted in 100 μL of acetonitrile (ACN). Standards for AEA, 2-AG, PEA, OEA, *N*-(2-hydroxyethyl)-9Z-octadecenamide), arachidonyl ethanolamide-d8 (*N*-(2-Hydroxyethyl)-5Z, 8Z, 11Z, 14Z-eicosatetraenamide-d8, AEA-d8) and 2-arachidonyl glycerol-d8 (2-AG-d8, (5Z, 8Z, 11Z, 14Z)-5, 8, 11, 14-Eicosatetraenoic acid-d8, 2-hydroxy-1-(hydroxymethyl)ethyl ester-d8) were purchased from Cambridge BioSciences, UK.

Following sample preparation, 10 μL of final sample extract was analyzed using liquid chromatography electrospray ionisation mass spectrometry (LC-ESI-MS/MS). The HPLC system used was a modular Shimadzu Vp series LC (Shimadzu, Milton Keynes, UK), with pumps, chilled autosampler and column oven. The HPLC column used was an ACE 3 C8 (100 × 2.1 mm, 3 mm) with guard column. The mobile phase A was water with 1 g/L ammonium acetate and 0.1% formic acid and mobile phase B was ACN with 1 g/L ammonium acetate and 0.1% formic acid pre-dissolved in 10% H_2_O. The flow rate was 300 μL/min. The MS system used was a SCIEX 4000 QTrap triple quadrupole mass spectrometer (Sciex, Warrington, UK) operated in electrospray positive multiple reaction monitoring mode. Quantification was performed using Analyst 1.6 and identification of each compound in plasma was confirmed by LC retention times of each standard and precursor and product ion *m/z* ratios. The peak area of each analyte is compared to a known amount of standard to determine the amount of target compound present.

2-AG in these samples were below the limit of quantification with our methodology in the plasma samples and the data has not been reported.

### Statistical Analysis

Data is presented as a scatter plot with mean ± SEM. Data sets were compared by paired Student’s *t*-test pre and post-activity. Correlations between plasma eCBs levels and hunger ratings, cardiovascular parameters or mood pre and post-activities were analyzed by linear regression. A quality control check was performed by a separate researcher on data entry.

## Results

All but one of the participants completed the study in full; one participant was unable to finish the cycling activity and did not have a final blood draw or complete the surveys. Thus nine participates were in the final comparison, except for the cycling activity where *n* = 8.

### Haemodynamics

**Figure 1:**
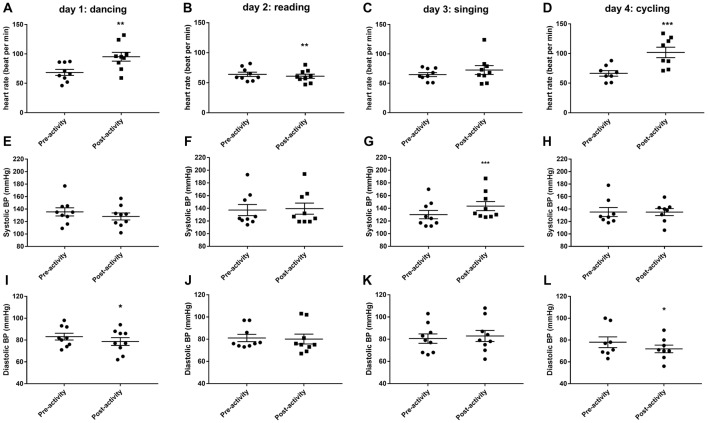
Changes in cardiovascular parameters (heart rate, HR; **A–D**), systolic blood pressure **(E–H)** and diastolic blood pressure **(I–L)** before and after 30 min activity (dancing, reading, singing or cycling) in nine healthy female volunteers. Data is presented as a scatter plot with mean ± SEM. Data sets were compared by paired Student’s *t*-test pre and post-activity (*P < 0.05, **P < 0.01 and ***P < 0.001).

Thirty minutes of dancing significantly increased HR (*t*_(8)_ = 4.894, *P* < 0.01, Figure 1A) and decreased diastolic blood pressure (*t*_(8)_ = 2.764, *P* < 0.05, Figure 1I). Thirty minutes of reading caused a small but significant reduction in HR (*t*_(8)_ = 3.736, *P* < 0.01, Figure 1B). Thirty minutes of singing increased systolic blood pressure (*t*_(8)_ = 5.66, *P* < 0.001, Figure 1G). Thirty minutes of cycling significantly increased HR (*t*_(7)_ = 7.314, *P* < 0.001, Figure 1D) and decreased diastolic blood pressure (*t*_(7)_ = 2.567, *P* < 0.05, Figure 1L).

### Hunger Scores

**Figure 2:**
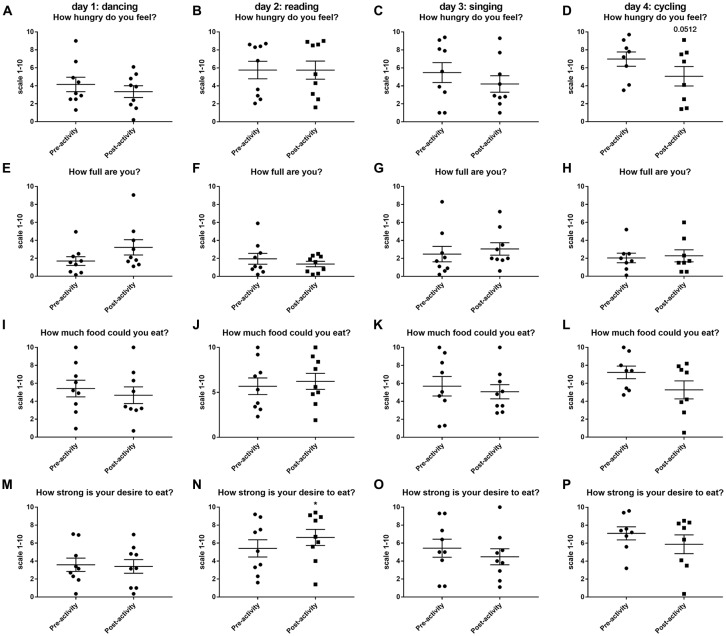
Changes in hunger and appetite scores as assessed using a visual analog scale (VAS) (1–10) before and after 30 min activity (dancing **(A,E,L,M)**, reading **(B,F,J,N)**, singing **(C,G,K,O)** or cycling **(D,H,L,P)** in nine healthy female volunteers. Data is presented as a scatter plot with mean ± SEM. Data sets were compared by paired Student’s *t*-test pre and post-activity (*P < 0.05).

The only significant change in hunger and appetite scores were observed after 30 min of reading when volunteers reported a significantly higher desire to eat (Figure 2N). Volunteers tended to have reduced hunger ratings after dancing, singing and cycling (Figure 2), but this only reached near significance for the question “how hungry do you feel?” immediate post-cycling (*t*_(7)_ = 2.348, *P* = 0.0512, Figure 2D).

### Mood Scores

**Figure 3:**
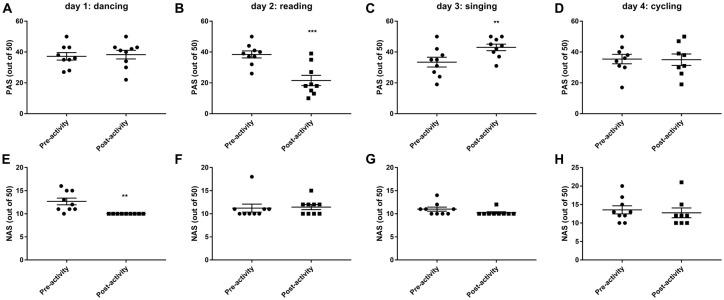
Changes in postive (PAS, **A–D**) and negative (NAS, **E–H**) mood and emotions before and after 30 min activity (dancing, reading, singing or cycling) in nine healthy female volunteers. Data is presented as a scatter plot with mean ± SEM. Data sets were compared by paired Student’s *t*-test pre and post-activity (**P < 0.01, ***P < 0.001).

Dancing decreased negative mood and emotions (*t*_(8)_ = 3.671, *P* < 0.01, Figure 3E), while reading decreased positive mood and emotions (*t*_(8)_ = 5.751, *P* < 0.001, Figure 3B). Only singing was found to significantly improve positive mood and emotions (*t*_(8)_ = 4.951, *P* < 0.01, Figure 3C) and also tended to decrease negative mood and emotions (eight out of nine volunteers reported a lower NAS post-singing, Figure 3E). Cycling has no effect on mood ratings.

### Plasma Levels of Endocannabinoids

**Figure 4:**
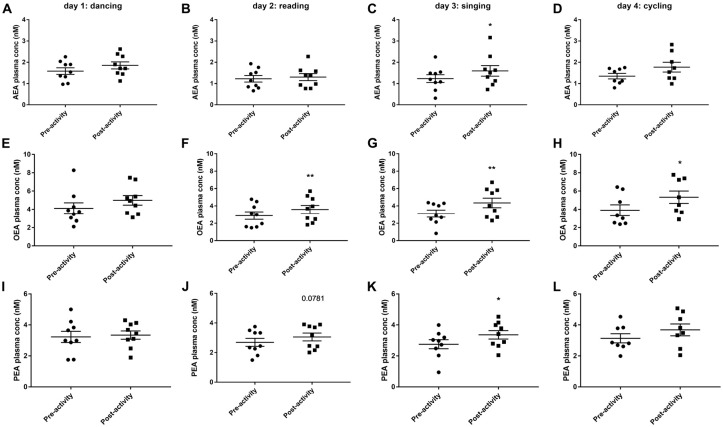
Plasma endocannabinoid levels (AEA, anandamide, **A–D**; OEA, oleoylethanolamine, **E–H**; PEA, palmitoylethanolamine, **I–L**) before and after 30 min activity (dancing, reading, singing or cycling) in nine healthy female volunteers. Data is presented as a scatter plot with mean ± SEM. Data sets were compared by paired Student’s *t*-test pre and post-activity (*P < 0.05, **P < 0.01).

Dancing had no effect on circulating levels of eCBs measured immediately the activity, although there was a trend for AEA and OEA levels to be increased (Figures 4A,E). Thirty minutes of reading significantly increased plasma OEA levels (*t*_(8)_ = 4.586, *P* < 0.01, Figure 4F) and tended to increase PEA levels (*t*_(8)_ = 2.02, *P* = 0.078, Figure 4J). Singing significantly increased the plasma levels of all eCBs measurable; AEA (*t*_(8)_ = 3.049, *P* < 0.05, Figure 4C), OEA (*t*_(8)_ = 4.81, *P* < 0.01, Figure 4G) and PEA (*t*_(8)_ = 3.319, *P* < 0.05, Figure 4K). OEA levels were also increased after 30 min cycling (*t*_(6)_ = 3.594, *P* < 0.05, Figure 4H).

At baseline (before activities started) across all 4 days, there was a significant positive correlation between plasma OEA levels and the rating for “how much food could you eat?” (*r*^2^ = 0.2226, *F* = 9.16, *P* < 0.01) and positive mood and emotions (*r*^2^ = 0.1355, *F* = 5.172, *P* < 0.05). Resting HR was positively correlated with both plasma AEA (*r*^2^ = 0.3363, *F* = 16.72, *P* < 0.001) and PEA (*r*^2^ = 0.169, *F* = 6.711, *P* < 0.05) levels.

**Figure 5:**
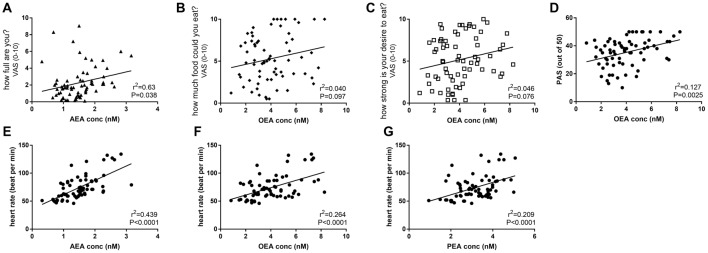
Correlations between plasma eCBs levels and appetite **(A–C)**, mood **(D)** and HR **(E–G)** in healthy female volunteers when measured pre or post-activities and analyzed by linear regression.

Across all days and time points (pre- and post-activity), plasma AEA levels were positively correlated with the rating for “how full are you?” (*r*^2^ = 0.0626, *F* = 4.472, *P* < 0.05, Figure 5A), and plasma OEA levels tended to be positively correlated with the rating for “how much food could you eat?” (*r*^2^ = 0.0404, *F* = 2.821, *P* = 0.097, Figure 5B) and “how strong is your desire to eat?” (*r*^2^ = 0.04624, *F* = 3.248, *P* = 0.076, Figure 5C) and with increased ratings for positive mood and emotion (*r*^2^ = 0.1269, *F* = 9.879, *P* < 0.01, Figure 5D). All three ethanolamines were positively correlated with HR (AEA: *r*^2^ = 0.4394, *F* = 53.3, *P* < 0.0001, Figure 5E; OEA: *r*^2^ = 0.2639, *F* = 24.37, *P* < 0.0001, Figure 5F and PEA: *r*^2^ = 0.2093, *F* = 18, *P* < 0.0001, Figure 5G).

## Discussion

It is well reported that running is correlated with mood elevation. These positive effects have been attributed to an evolutionary trait, where positive re-enforcement ultimately led to increased food foraging, survival and subsequent passing of relevant genes to offspring and have recently been attributed, at least in part, to increases in eCBs (Bramble and Lieberman, 2004; Raichlen et al., 2012). Our study aimed to examine whether activities other than running also increase eCBs and enhance mood. We have shown for the first time that singing significantly increases levels of AEA, OEA and PEA in healthy post-menopausal females and enhanced mood. Dancing (on mood) and cycling (on eCBs) also had positive effects in this group. Although singing was the most beneficial activity in this study, this is likely to reflect the fact that the volunteers were recruited from local choirs and already find this an enjoyable activity. These data provide biochemical evidence of an increase in novel signaling messengers known to improve mood, reduce stress and anxiety, enhance memory, protect brain function and reduce pain.

Singing, in particular group singing, has been associated with an increase in positive mood and improved immune function in humans (Kreutz et al., 2004; Schladt et al., 2017). Choir singing also enables social interactions, exhibiting a greater benefit to mood than singing alone (Schladt et al., 2017). Our results also demonstrate that singing increases mood, and also for the first time that singing increasing circulating levels of AEA, OEA and PEA. As AEA is a partial agonist of CB_1_ and has full agonist activity at TRPV1, an increase in the levels of AEA post activity could therefore facilitate increases in positive emotions, as well as anxiolytic and analgesic effects (Chapman et al., 2009; Starowicz et al., 2012). Levels of OEA post activity were correlated with a decrease in hunger and desire to eat. This supports previous data that OEA attenuates food consumption and increase lipolysis and energy expenditure (Lo Verme et al., 2005a,b). *In vivo* studies conducted in mice have also suggested beneficial neuroprotective effects of OEA, this protective effect could potentially be translated to humans and warrants further study (Galan-Rodriguez et al., 2009; Zhou et al., 2012; Yang et al., 2015). An abundance of evidence has supported PEA as a potential therapy for neurological and inflammatory disorders, particularly those associated with pain (Costa et al., 2008; Keppel Hesselink, 2012; Esposito and Cuzzocrea, 2013). PEA has also been taken into clinical trials, whereby 600 mg of PEA was shown to be effective in various pain states, without exhibiting any safety issues (Hesselink and Hekker, 2012). Therefore, it could be beneficial to increase levels of PEA via activities such as singing, to promote neuroprotection, analgesia and reduce inflammation. It is also important to note that increasing OEA and PEA can indirectly increase AEA responses by the entourage effect by competitive inhibition of AEA degradation by fatty acid amide hydrolase (FAAH; Di Marzo et al., 2001; Costa et al., 2008; Ho et al., 2008). Overall, singing could be a valuable activity in patient populations that suffer with dysfunctions in psychological well-being and struggle to participate in aerobic/moderate intensity exercise.

Cycling resulted in a significant increase in OEA, and in a trend for increases in both AEA and PEA. These changes corresponded with a decrease in participants desire to eat and how hungry they felt. These data are consistent with results from a previous study where plasma OEA levels were significantly increased after 30 min of cycling in 16 male non-smokers with a mean age of 22.9 years (Cedernaes et al., 2016). Our data did not show that cycling positively affected mood (no increase in PAS or decrease in NAS). Brellenthin et al. (2017) showed that acute aerobic exercise (both prescribed and preferred) resulted in positive mood outcomes in individuals capable of a range of levels of physical activity, as well as showing modulation of the eCB system. Interestingly, the group that undertook their “preferred exercise” had the best effect in reducing anxiety and improving mood. In the present study, singing by participants recruited from a choir support these observations, suggesting that the eCB system is not only responsible for the motivation for exercise (i.e., reward driven), but also the pleasure associated with an activity that an individual enjoys. It would also have been interesting if another group had been included (i.e. not recruited from a choir) to directly assess the concept of preferred vs. prescribed activity and to confirm that carrying out an activity that is “pleasurable” to an individual is an important factor in the psychological benefits of exercise and other related activities. Subjects were also not asked to rate how much they enjoyed each of the activities, this would have been an interesting endpoint to assess to what degree the participant’s moods were influenced by how much they liked a particular activity and should be considered in future study. These factors would also have provided further evidence to why individuals in this study failed to experience positive mood changes or significant increases in AEA post cycling; compared with the study by Heyman et al. (2012) where increases in AEA were seen in well-trained cyclists, who presumably enjoy cycling.

Exercise intensity may be another factor explaining the lack of AEA increases in our participants. Brellenthin et al. (2017) showed that the greatest increases in 2-AG and AEA were seen in the higher intensity exercise group. Sparling et al. (2003) also showed significant increases in AEA when participants reached 70%–80% max HR. According to Gulati et al. (2010), maximum HR for women is calculated as 206−(0.88 × age of patient). As the average age of our participants was 61 years, their average maximum HR (max HR) is approximately 154 bpm, meaning their 70%–80% max HR should be 107–123 bpm. Cycling was the only activity that almost reached this (average 102 bpm immediately post exercise) and dancing resulted in an average HR of 95 bpm (immediately post exercise). This could suggest that our activities may not have been intense enough to elicit significant changes in circulating eCBs.

A number of studies have shown that dance is an effective therapy in improving mood (including mild depression), enhancing social interactions, boosting self-confidence, as well as improving physical activity (Akandere and Demir, 2011; Kiepe et al., 2012; Meekums et al., 2015). In one study, dancing caused an increase in plasma serotonin levels and a decrease in negative psychological symptoms in a group of 20 female adolescents with mild depression, compared to 20 control subjects (Jeong et al., 2005). We found post activity that there was a significant decrease in negative emotions following 30 min of dancing. It should be acknowledged that the decrease in negative emotions could also be because this was the activity undertaken on day 1 and participants had higher NAS scores before starting the study. Although there was a trend in increasing levels of AEA and OEA levels post-activity, this did not reach significance. Our results suggest that dancing did not effectively increase eCB levels or improve mood, however this could be because they were unfamiliar with the class, therefore not finding it as enjoyable as singing as this was more familiar to them, or that the class was not at a high enough intensity to produce changes in eCB levels. It should also be noted that our participants were older than those previously studied, and there could be an age-related decline in the eCB response to exercise.

Reading was used as a control activity to assess baseline eCB levels and mood. We found that reading was the only activity that increased participants desire to eat but had little impact on overall fullness or actual hunger and was correlated to increases in OEA post activity. Reading also decreased the ratings for positive mood and emotions. In hindsight, because subjects were unaware of the task, the activity set-up looked like they were about to take an exam, which may have resulted in unforeseen heightened anxiety levels. Recent studies have implicated the eCB system as a possible mediator of hedonic vs. homeostatic eating response to the consumption of food (as a reward) as well as acute stress and anxiety (Matias et al., 2006; Monteleone et al., 2015, 2017). Dlugos et al. (2012) showed that AEA, PEA and OEA were all increased in serum in response to stress. They also found that higher levels of AEA at baseline, associated with decreased levels of anxiety. Furthermore, a common phenomenon is that typically negative emotions, particularly boredom, stress and depressive emotions increase our desire to eat in order to increase positive emotions (Koball et al., 2012; Yau and Potenza, 2013; Moynihan et al., 2015). These factors could explain the elevated levels of OEA post activity and lower PAS scores.

A limitation of our study is that participants already had very low negative affect scores and high positive affect scores. This suggests that the individuals that took part in the study were generally happy and positive and there was therefore little room for mood to be further improved. It would therefore be interesting in future work to see the effects of these same activities on individuals that exhibit depressive, or anxious behavior in order to see greater differences in negative emotional responses. Intensity of a physical activity has also been shown to influence exercise induced increases in eCB levels. Raichlen et al. (2013) built on previous work showing that eCBs follow a U-shaped curve, with moderate level activity resulting in the biggest increase in eCB levels (Berger and Motl, 2000). This trend in eCB levels is also correlated with mood as the positive emotional state post exercise is not experienced at very low or very high intensities (Berger and Motl, 2000). As all the participants were unfamiliar to the activities they carried out, a lot of their focus would have been on “mastering” the class rather than actually enjoying it in the moment.

It can also not be overlooked that this study only recruited healthy female volunteers. Evidence from animal studies has already shown distinct sexual dimorphism in the eCB system, particularly in CB_1_ expression and activation (Reich et al., 2009; Mateos et al., 2011; Dias-Rocha et al., 2018). Limited preliminary evidence from human studies have also shown variations in the eCB system between males and females (Cupini et al., 2006; Hill et al., 2008). Thus future study should look to establish whether the effects observed in this study translate to male participants as well as females.

In conclusion, we found that activities other than running (singing, dancing and cycling) can increase plasma eCB levels. Singing was the only activity to increase plasma levels of AEA and improve positive mood outcomes, suggesting that singing in this group of volunteers was able to produce an endogenous “high.” This is interesting as the participants were recruited from a choir, suggesting that the enjoyment of an activity may influence their feeling of reward and the eCB response. This preliminary evidence suggests that activities like singing could be recommended to individuals suffering from mood disorders such as anxiety and depression, as well as a potential therapy for neurological and inflammatory disorders. Future research should consider an individual’s preference to a particular activity, as this could be an important factor in influencing the eCB system, as well as being a factor in deciding appropriate therapy.

## Author Contributions

NS and SO’S wrote the article with contributions from all the other authors. SO’S, SM and NS carried out cardiovascular measurements, surveys and blood processing. SO’S processed the study data and performed the statistical analysis. CO and DB performed the eCB analysis on the plasma samples. PH carried out the blood draws from the subjects. VM developed the study with SO’S.

## Conflict of Interest Statement

The authors declare that the research was conducted in the absence of any commercial or financial relationships that could be construed as a potential conflict of interest.

## Abbreviations

2-AG: 2-arachidonoylglycerol
AEA: anandamide
BBB: blood brain barrier
BDNF: brain derived neurotrophic factor
eCBs: endocannabinoids
LC-ESI-MS-MS: electrospray ionization liquid chromatography/mass spectrometry
OEA: oleoylethanolamine
PEA: palmitoylethanolamine.
